# Integration of Facial Expression and Gaze Direction in Individuals with a High Level of Autistic Traits

**DOI:** 10.3390/ijerph19052798

**Published:** 2022-02-27

**Authors:** Andrea Marotta, Belén Aranda-Martín, Marco De Cono, María Ángeles Ballesteros-Duperón, Maria Casagrande, Juan Lupiáñez

**Affiliations:** 1 Department of Experimental Psychology and Mind, Brain and Behavior Research Center (CIMCYC), University of Granada, 18071 Granada, Spain; jlupiane@ugr.es; 2 Department of Psychobiology and Mind, Brain and Behavior Research Center (CIMCYC), University of Granada, 18071 Granada, Spain; marco.decono1994@gmail.com (M.D.C.); maballes@ugr.es (M.Á.B.-D.); 3 Dipartimento di Psicologia Dinamica e Clinica, Sapienza Università di Roma, 00185 Rome, Italy; maria.casagrande@uniroma1.it

**Keywords:** eye-gaze, emotional expression, autistic traits, congruency effect

## Abstract

Background. We investigated whether individuals with high levels of autistic traits integrate relevant communicative signals, such as facial expression, when decoding eye-gaze direction. Methods. Students with high vs. low scores on the Autism Spectrum Quotient (AQ) performed a task in which they responded to the eye directions of faces, presented on the left or the right side of a screen, portraying different emotional expressions. Results. In both groups, the identification of gaze direction was faster when the eyes were directed towards the center of the scene. However, in the low AQ group, this effect was larger for happy faces than for neutral faces or faces showing other emotional expressions, whereas participants from high AQ group were not affected by emotional expressions. Conclusions. These results suggest that individuals with more autistic traits may not integrate multiple communicative signals based on their emotional value.

## 1. Introduction

Faces are among the most important visual stimuli, conveying complex information of considerable importance in the context of social interactions, including identity, race, sex, attractiveness, and emotions [1,2,3]. Humans have a marked preference for real face visual features and face-like configurations very early in their ontogeny. Infants, and even fetuses, show a visual preference for basic face-like configurations [4,5,6]. This preference is very functional to the newborn, facilitating connection with the caregiver and evoking a response [7]. Infants use a wide variety of social signals to respond contingently to their social partner, for instance, coupling their facial movements and vocalizations with the facial expression of their caregivers [8,9]. The preference for face visual features has also been observed in school-aged children [10,11,12] and in adults [13,14,15]. For example, masked faces are detected more quickly and accurately than masked objects [16], and facial changes are better detected than changes in non-facial objects [17].

Among the changeable aspects of the face, gaze shifts and facial expressions are crucial, as they provide humans with powerful social signals that allow to infer internal states and intentions. Eye-gaze direction signals another person’s focus of interest and can orient our attention to potentially relevant locations or objects in the surrounding space [18,19]. When interpreting eye-gaze direction, people consider information from different sources, such as the iris/sclera ratio [20], the head posture [21], the presence of object near the fixation point of another person’s [22] and, of relevance for the present study, the emotional facial expression.

Facial expressions of other people can help to determine the emotional state or motivational intentions, and several pieces of evidence indicate that the processing of gaze direction and emotional expression mutually interact. 

On the one hand, some studies have observed that gaze direction can modulate the time to judge facial expressions. For example, faces expressing anger or joy are recognized more quickly when presented with gaze directed at the viewer than when presented with gaze averted. Contrastingly, sadness and fear are recognized faster with averted gaze [23,24]. Adams and Kleck interpreted these findings in terms of a shared signal hypothesis, in which happiness and anger are considered ‘approach-oriented’ emotions and sadness and fear ‘avoidance-oriented’. 

On the other hand, the perception of gaze direction is modulated by emotional expressions. For example, Lobmaier and Perrett (2011) [25] asked participants to judge whether faces presented in different orientations and with different facial expressions were looking towards them. They found that smiling faces are more likely to be interpreted as directed towards the observer than fearful, angry, and neutral faces. These findings are not consistent with the shared signal hypothesis, and they have been explained by the “self-referential positivity bias” hypothesis [26], according to which people are more likely to believe that they are the source of someone else’s happiness, so as to improve self-esteem.

Reduced interest in the human face and malfunctioning of the above-described face-related attentional processes represent some of the most pronounced social deficits associated with autism spectrum disorder (ASD) [27,28,29]. A large amount of empirical evidence has highlighted the presence of an atypical imbalance in attention to social versus non-social stimuli in ASD [30]. Children and adults with ASD exhibit poorer recognition memory for faces and reduced visual attention to facial stimuli than typically developing (TD) individuals [31,32]. Neurophysiological evidence also corroborated the presence of abnormal facial processing in ASD. For example, recent studies combining EEG and eye-tracking measures have observed a reduction in social bias and an abnormal orientation to faces in individuals with ASD [33,34]. Eye-tracking research has also shown that a reduced fixation to the eye area in ASD [35] can result in significant differences in brain activation. For instance, people with ASD showed greater activation in the social neural network to averted than to direct gaze, this pattern being the opposite of that observed in TD [36]. Nevertheless, those differences do not seem to affect the interpretation of gaze direction and object detection but rather the ability to infer gaze intentionality [37].

Indeed, some studies have shown that people with ASD are equally adept at correctly identifying the direction of a gaze as TD individuals [38,39,40,41]. However, they seem to present difficulties in integrating gaze direction with communicative and social contexts [42,43]. In particular, of relevance for the present study, Akechi et al. [44] observed that autistic children had a deficit in integrating the information of facial expressions with eye-gaze direction.

Additionally, several research studies have suggested that ASD represents the upper extreme of a pattern of social–emotional and communicative traits continuously distributed in the general population [45,46,47]. Initial support comes from studies demonstrating that the degree of autistic traits measured by the Autism Spectrum Quotient (AQ) [48] in a typical population is related to performance on behavioral tasks that show impairments in ASD, such as the ability to draw mentalistic inferences from the eyes [49] (Baron-Cohen et al., 2001a), the identification of emotional facial expressions [50], and the attentional cueing from eye gaze [51,52].

The main aim of the present study was to investigate whether individuals with high levels of autistic traits integrate facial expression when decoding eye-gaze direction. As mentioned above, the combination of expression and gaze direction provides essential information for understanding another individual’s intentions, and difficulties in their encoding and integration have been observed in ASD. However, it is not clear whether this impairment is directly related to autism traits per se or rather depends on different social communication patterns formed by years of altered social experience. Testing individuals who function normally in their everyday lives and who do not usually avoid social contacts would allow testing the specific contribution of autistic traits, minimizing the influence of experience, such as the amount of social involvement. 

To achieve this aim, we used the gaze discrimination task developed by Cañadas and Lupiáñez (2012) [53] to explore the importance of eye-gaze direction in spatial interference paradigms. These authors demonstrated that gaze direction discrimination of a lateralized face (i.e., presented to the left of right of fixation point) is faster and more accurate when the gaze is oriented inwards, towards the center of the scene (e.g., right averted gaze presented on the left) than when it is directed outwards (e.g., right averted gaze presented on the right). This effect was opposite that of the classical results generally observed with non-social stimuli, such as arrows (e.g., faster reaction time for arrows pointing outwards) [54], and it was interpreted in terms of eye contact (e.g., a speeding up of responses when the target face seems to look directly at the participants). A further investigation revealed that the emotional expression of the face modulated the inward effect [55] according to the “shared signal hypothesis” [33,34]: the effect was larger when it was coupled with approach-oriented emotions such as happiness and anger, while it was smaller for the avoidance-oriented emotions such as fear. 

The predictions of the present study were straightforward. If the degree of autistic traits in typical population is related to the difficulties in integrating gaze direction with communicative and social contexts generally observed in ASD, then these difficulties should be observed only in participants with high levels of autistic traits but not in participants with low autistic traits. In other words, the identification of gaze direction should not be affected by the emotional expression of facial stimuli in participants with high levels of autistic traits, while the inward effect from gaze direction should be modulated in participants with low levels of autistic traits. 

Moreover, there are two possible scenarios regarding the modulation of facial expressions on the identification of gaze direction in participants with low autistic traits. If the emotional expression modulates the identification of gaze direction, according to the shared signal hypothesis [33,34], then a larger inward effect should be observed with both happy and angry faces (approach-oriented emotions) as compared with sad and fearful faces (avoidance-oriented emotions). In contrast, if the identification of gaze direction is modulated by emotional expression according to the “self-referential-positivity bias hypothesis” [25,56], then the inward effect should be larger for happy faces than for faces showing other emotional expressions or a neutral expression.

## 2. Methods

### 2.1. Participants

Initially, 459 students completed the AQ (mean (M) score = 16.29; standard deviation (SD) = 5.88). Next, 36 students from the upper and lower quartiles of the AQ distribution (using cutoff scores equal to or lower than 11 and scores equal to or higher than 22) were invited to complete further testing. Based on previous research [57,58], we decided to use permissive cutoff scores for reasons related to sample size since only 1.74% of the initial group would have met the clinical AQ cutoff scores of 32 [48]. All participants from the initial group were undergraduate psychology students. Women were overrepresented in the initial group (80%) and the sample selected for this study (86.11%). The characteristics of the high and low AQ groups are outlined in Table 1. The groups did not differ significantly in age distribution, *F*(1,34) < 1. Numerically, there was a higher proportion of females in the high AQ group than in the low AQ group, but this difference was not statistically significant, χ^2^(1) = 2.09, *p* = 0.148. There were no significant socio-demographic (e.g., education, ethnic origin, native language) differences between these two groups.

### 2.2. The Autism Spectrum Quotient (AQ)

The AQ is a 50-item self-report questionnaire designed for measuring autistic traits in the general population [48]. In particular, it assesses five different domains relevant for autistic traits: social skills, attention to detail, attention switching, communication, and imagination. This instrument (retrieved from https://www.autismresearchcentre.com/arc_tests (accessed on 8 April 2018) has been used specifically for quantifying where participants are situated on the continuum from autism to normality. The AQ score has been shown to have good test–retest reliability, good internal consistency, and acceptably high sensitivity and specificity [48].

### 2.3. Apparatus and Stimuli

The E-Prime 2.0 software (Psychology Software Tools Inc, Pittsburgh, PA, USA) was used to control stimuli presentation, timing, and data collection. Stimuli were presented on a 17″ screen running at a 1024 × 768 pixel resolution. They consisted of 40 full-color photographs of four males and four females (dimensions = 180 pixels × 200 pixels or 6.67° × 5.72°) displaying either a neutral, angry, sad, fearful, or happy emotional expression. Faces were selected from the Karolinska Directed Emotional Faces [59] and were manipulated with Adobe Photoshop CS6 to change gaze directions to the left and right sides.

### 2.4. Procedure

**Figure 1:**
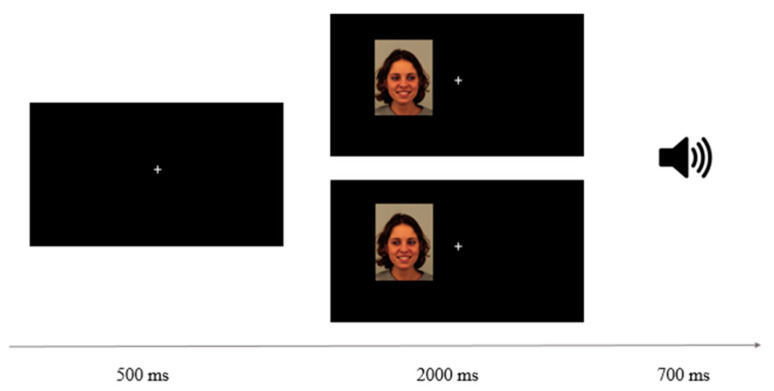
Schematic view of a trial sequence from left to right. The examples illustrate, from top to bottom, an inward and an outward trial of a woman with an emotional expression of happiness. The speaker icon represents the auditory feedback given on incorrect answers.

Gaze Discrimination Task. Participants were required to discriminate, as fast and accurately as possible, the direction (left or right) of the eye gaze of the faces presented to the right or the left of a fixation point. They were tested while seated at approximately 60 cm away from the monitor in a faintly lit room. Each trial started with the onset of a white fixation cross (0.5° × 0.5°) centered on a black computer screen for 500 ms. Then, a face displaying different emotional expressions, was presented either to the right or left of the fixation cross and gazing either to the right or left (see Figure 1). The distance from the inner edge of the face to the central fixation was approximately 3.02°. Importantly, this design produced inward trials where eyes were directed towards the central fixation location (i.e., a right-averted gaze of faces presented on the left, and a left-averted gaze of faces presented on the right) or outward trials (i.e., a left averted gaze presented on the left and a right averted gaze presented on the right). Participants had to discriminate the gaze direction of the face by pressing the “Z” or “M” key of the computer keyboard when the correct answer was left or right, respectively. Feedback on no-response or incorrect response trials was provided via a 220 Hz tone for 700 ms. All possible combinations of stimuli, 8 (face identity) × 5 (emotional expression) × 2 (presentation side) × 2 (gaze direction), formed a total of 160 trials. Two blocks of trials with all combinations were presented for a total of 320 trials. Participants completed a practice block of 16 randomly selected trials to familiarize themselves with the task, followed by eight experimental subblocks of 40 randomly selected trials each, with a rest period between blocks.

Emotional Expression Categorization Task. After completing the gaze discrimination task, participants were prompted to pay attention to the screen for one last activity, in which they had to identify the emotional expressions of the same faces presented in the previous task. Faces with a direct gaze displaying either angry, happy, sad, neutral, or fearful emotional expression appeared at the center of the screen for an unlimited time. Participants were required to identify the expression of faces by typing the answer on a computer keyboard.

### 2.5. Design

A 2 (Group: high AQ vs. low AQ) × 5 (Emotional Expression: happy, angry, fearful, neutral, or sad) × 2 (Gaze: inward trials vs. outward trials) mixed design was used to analyze the data. The extent of AQ traits was treated as a between-participants variable, and emotional expression and gaze direction represented within-participants factors. Two univariate analyses of variance (ANOVA) separately considered mean corrected RTs and percentage of errors as dependent variables. If the relevant high-order interactions were significant, the inward effect will also be calculated (inward trials—outward trials) and used as a dependent variable in the Bonferroni post hoc testing. As in the Cañadas and Lupiáñez study (2012) [53], trials with reaction times (RTs) faster than 200 ms (0.2% of the trials) or slower than 1300 ms (0.8% of the trials) were considered anticipations and lapses, respectively, and were excluded from the RTs analysis, together with incorrect responses (5.5% of the trials). Mean RTs were computed for each experimental condition using the remaining observations (see Table 2).

## 3. Results

### 3.1. Gaze Discrimination Task

**Figure 2:**
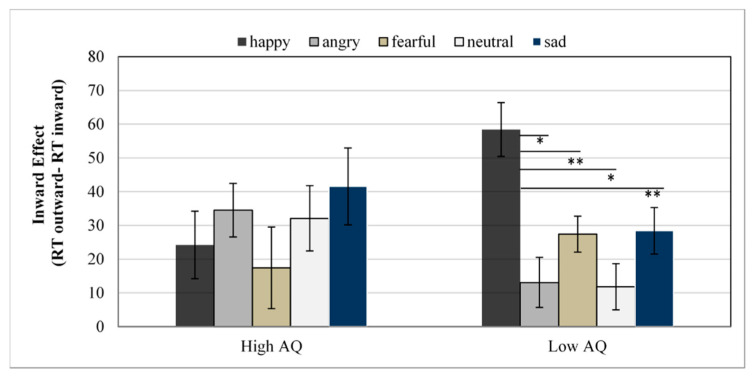
Mean reaction times for the inward effect (RTs differences between outward and inward trials) as a function of emotional expressions and group. Error bars represent the standard error of the mean for each condition. * = *p* < 0.05; ** = *p* < 0.001.

Reaction Times. The ANOVA revealed a significant main effect of Emotional Expression, *F*(4,136) = 13.68, *p <* 0.001, *η*^2^_p_ = 0.29, with lowest reaction times for fearful faces (617 ms) and highest for angry faces (645 ms). The main effect of the Group was not significant, *F*(1,34) = 2.16, *p* = 0.150. A main effect of Gaze was also found, *F*(1,34) = 48.85, *p* ˂ 0.001, *η*^2^_p_ = 0.59, with faster RTs for inward (616 ms) than for outward trials (645 ms). Furthermore, significant interactions were observed between Emotional Expression and Gaze, *F*(4,136) = 3.14, *p* = 0.016, *η*^2^_p_ = 0.08, and between Group, Emotional Expression, and Gaze, *F*(4,136) = 5.84, *p* ˂ 0.001, *η*^2^_p_ = 0.15 (see Figure 2). Emotional Expression × Gaze ANOVAs were conducted separately for each Group, showing that the interaction was only significant in the low AQ group, *F*(4,68) = 7.12, *p* ˂ 0.001, *η*^2^_p_ = 0.29. Post hoc test using the Bonferroni correction revealed an inward gaze effect (RT outward trials—RT inward trials) significantly larger for happy faces compared with neutral (mean difference = 46.61, *p* ˂ 0.001), anger (mean difference = 45.33, *p* ˂ 0.001), fear (mean difference = 31.00, *p* = 0.03), or sadness (mean difference = 29.99, *p* = 0.04) faces, as shown in Figure 2. No other significant difference between emotions was found. In the high AQ group, the Emotional Expression by Gaze interaction was not significant, *F*(4,68) = 1.81, *p* = 0.137, *η*^2^_p_ = 0.09. Post hoc analysis revealed no difference between any emotion (all *p* > 0.05).

Errors. The ANOVA revealed a significant main effect of Emotional Expression, *F*(4,136) = 5.56, *p* ˂ 0.001, *η*^2^_p_ = 0.14, with the lowest percentage of errors being for fearful faces (4.1%) and the highest for angry faces (6.9%). A main effect of Gaze was also found, *F*(1,34) = 7.40, *p* = 0.01, *η*^2^_p_ = 0.18, with a lower percentage of errors for inward trials (4.2%) than for outward trials (6.3%). The main effect of Group was not significant, *F*(1,34) = 1.53, *p* = 0.224, *η*^2^_p_ = 0.04. The Emotional Expression x Group interaction was also significant, *F*(4,136) = 2.80, *p* = 0.028, *η*^2^_p_ = 0.08. Post hoc test using the Bonferroni correction showed that the Low AQ group committed more errors responding to angry faces than happy (mean difference = 3.28, *p* = 0.05) and neutral ones (mean difference = 3.33, *p* = 0.04). However, the high AQ group committed more errors responding to happy faces than to fearful ones (mean difference = 3.47, *p* = 0.027). No other differences between groups or emotions were found. No other interaction reached significance.

### 3.2. Emotional Expression Identification Task

For the analysis of responses in the emotional expression task, a 2 (Group: high AQ vs. low AQ) × 5 (Emotional Expression: happy, angry, fearful, neutral, or sad) mixed design was conducted with the accuracy of responses as the dependent variable. The analysis of accuracy data indicated a main effect of emotional expression, *F*(4,136) = 2.80, *p* < 0.001, *η*^2^_p_ = 0.48. The highest accuracy was observed for faces displaying happiness (97.9%) and the lowest for neutral faces (45.1%). Importantly, the results show that Group did not have any effect, *F* < 1, and did not modulate the effects of emotional expression, *F* < 1.

## 4. Discussion

The present study examined the modulation of people’s autistic traits in the discrimination of the eyes direction displayed by faces expressing different emotions. In particular, we aimed to investigate whether the ability to integrate facial expression when decoding eye-gaze direction depended on the extent of autistic-like traits measured with the AQ. Both low and high AQ groups showed that the identification of gaze direction was faster when the eyes were directed towards the center of the scene (inward effect). It is interesting that participants from the high AQ group also showed this effect, as it has been recently shown that it is not observed until late childhood, with 4-year-old children showing instead a similar effect for gaze and arrows in this task [60]. 

However, this effect was mediated by facial expression only in the group with low autistic traits, so the inward effect was larger for happy faces than for neutral faces or faces showing other emotional expressions. This finding contrasts with the pattern of results observed by Jones [55] in the general population.

Consistent with the shared signal hypothesis, Jones showed larger inward effects when faces displayed approach-oriented emotions and smaller effects with avoidance-oriented emotions. In contrast, our findings revealed a distinction between negative and positive emotions and are more coherent with the self-referential-positivity bias hypothesis, according to which people prefer to interpret positive emotions as being directed towards them and negative facial expressions as directed away, in order to enhance self-esteem (cf. [25,56]). However, it is important to note that the shared signal hypothesis has been generally used to explain how the recognition of emotional expressions is affected by eye gaze direction [33,34], while the self-referential-positivity bias hypothesis has been generally used to explain how the identification of gaze direction is affected by emotional expression [25,56]. In our study, participants were required to identify gaze direction (left or right) of faces with different emotional expressions. In line with the self-referential-positivity bias predictions, we observed that the inward effect was larger for happy faces than for neutral faces or faces showing other emotional expressions in the AQ Low group.

There are some discrepancies between Jones’ study and ours. For example, Jones [55] (2009) used four facial expressions (happy, angry, fearful, and neutral), while our faces showed happy, angry, fearful, sad, and neutral expressions. Second, our participants were selected according to their autistic traits scores (e.g., students from the upper and lower quartiles of the AQ distribution), while in the Jones’ study, participants were recruited from the general population and were not selected based on their autistic traits. Therefore, it can be suggested that the different results of our study compared with Jones’s can be traced back to the autistic traits, high or low. However, future studies are needed to fully elucidate the reasons for these different findings. 

Of relevance, data from the present study further show that participants with high levels of autistic traits were not affected by the facial expression of the stimuli when decoding eye-gaze direction. Note that the current finding cannot be explained by a general difficulty of individuals with high levels of autistic traits in decoding eye-gaze direction because in the gaze discrimination task we did not find any group differences in overall accuracy or RTs. At the same time, we found no group differences in overall accuracy of the facial emotion identification task, which suggests that the absence of interaction between emotional expression and gaze direction in participants with high levels of autistic traits cannot be attributed to a general difficulty in decoding facial emotional expressions. Our findings are in line with studies showing that autistic individuals have difficulties integrating communicative signals present in the eyes with social and communicative contexts [42,43] and with their emotional value [44]. A possible explanation for why participants with high levels of autistic traits do not show a greater inward effect for happy faces may be related to a weaker self-referential-positivity bias of this group as compared with the low AQ group of participants. This is in line with previous research suggesting reduced positive self-evaluations in ASD [61,62], and it is coherent with previous research showing that judgments of persons with ASD appear to be less strongly modulated by the emotional value of the available information [63]. Another explanation may also be that these results are related to a decreased reward in the social interaction of individuals with high AQ. For this reason, they would appear to be less influenced by happy faces than people with low AQ. This corroborates previous evidence of a reduced reward value of smiling faces in autistic people compared with typical individuals [64]. Finally, the lack of integration between eye-gaze direction and emotional expressions may be based on perceptual or cognitive style, such as weak central coherence [65], which is not specific to the social domain. Individuals with ASD exhibit weak central coherence [66], which is an impaired ability to integrate individuals’ features into a coherent percept. If this impairment extends to those with high ASD traits, this could have impeded the integration of gaze direction and facial expression observed in our study. 

To summarize, findings from the present study suggest that the integration between facial expression and gaze direction may be absent or reduced in individuals with a high level of autistic traits. Emotional expression is a key factor when interpreting eye-gaze direction. For instance, smiling faces are more likely to be interpreted as being directed towards the observer than fearful, angry, and neutral faces [25,26]. Our results indicate that this is not the case for individuals with high levels of ASD traits. Previous data using different emotional tasks suggested that ASD participants were not affected by gaze direction when recognizing facial expressions [44]. We report for the first time that individuals with high ASD traits display a similar absence of integration between these two types of social cues. In particular, in our study, the inward effect elicited by the gaze direction was not affected by the face’s emotional expression. This extends the cognitive socio-emotional similarities between ASD and individuals with high autistic traits and contributes to the dimensional view of ASD as the pathological extreme of a phenotype continuously distributed in the general population. Further studies are needed to investigate the ability of individuals with ASD to encode and integrate non-verbal social cues, which could reveal the source of communication and social interaction difficulties in ASD.

## Figures and Tables

**Table 1:** Male–female ratio and means (and SDs) for AQ score and age.

Variables	Low AQ				High AQ	
	Mean	SD	N	Mean	SD	N
Gender (male/female)			1:17			4:14
Age (years)	18.89	1.6		20.44	3.01	
AQ score	8.28	1.84		24.61	3.18	

**Table 2:** Mean correct reaction times (RTs, in milliseconds), standard deviations (SDs), and percentages of incorrect responses errors (%IR) as a function of Emotional Expression, Gaze, and Group (high and low scores on AQ).

		Low AQ				High AQ	
Emotions	Gaze	RTs	SDs	%IR	RTs	SDs	%IR
Happy	Outward	643.4	73.03	5.579	662.8	84.62	10.77
	Inward	585.0	65.55	3.000	638.6	95.18	5.33
Angry	Outward	632.1	84.27	8.684	682.3	95.95	9.278
	Inward	619.0	74.56	6.632	647.8	91.05	4.83
Fearful	Outward	610.3	67.23	3.842	645.6	86.67	6.44
	Inward	582.9	65.70	4.105	628.1	88.33	2.72
Neutral	Outward	614.2	63.59	3.842	661.5	86.96	5.83
	Inward	602.4	78.22	3.895	629.4	94.92	4.167
Sad	Outward	624.6	72.24	5.053	670.0	95.25	6.77
	Inward	596.2	78.37	4.947	628.5	80.85	4.61

## Data Availability

The data that support the findings of this study are available on request from the corresponding author.
